# Individual Alcohol Consumption Depending on Regional Living Conditions: Results of a Russian Nationwide Study Based on 2012-2022 Data

**DOI:** 10.34172/jrhs.11352

**Published:** 2025-10-18

**Authors:** Sergey A. Maksimov, Svetlana A. Shalnova, Yulia A. Balanova, Asia E. Imaeva, Marina B. Kotova, Daria A. Kashtanova, Anna V. Kontsevaya, Oksana M. Drapkina

**Affiliations:** ^1^National Medical Research Center for Preventive Medicine, Moscow, Russia; ^2^Center for Strategic Planning and Management of Biomedical Health Risks, Moscow, Russia

**Keywords:** Alcohol drinking, Binge drinking, Population characteristics, Socioeconomic factors, Geographic locations, Russia

## Abstract

**Background:**

Individual alcohol consumption depends on living conditions at different territorial and environmental levels. This study examined the influence of regional living conditions on individual alcohol consumption based on the results of a large Russian nationwide study (2012-2022).

**Study Design:**

A cross-sectional multicenter observational study.

**Methods:**

Individual data from three stages of the Russian nationwide study, including the Epidemiology of Cardiovascular Diseases and Their Risk Factors in the Regions of the Russian Federation (ESSE-RF1) (2012-2014), ESSE-RF2 (2017-2018), and ESSE-RF3 (2020-2022), were used for investigation. The study samples included 53,902 men and women aged 25–74 years from 31 regions. Individual data were combined with the annual values of four regional indices that characterize economic, demographic, social, and industrial environmental conditions. The analyzed outcomes included any alcohol consumption and binge drinking.

**Results:**

The industrial development of regions was associated with an increased likelihood of any alcohol consumption (odds ratio [OR]: 1.66, 95% confidence interval [CI]: 1.59–1.72) and binge drinking (OR: 1.31, CI: 1.22–1.40). Improved economic (OR: 0.75, 95% CI: 0.72–0.78), demographic (OR: 0.73, 95% CI: 0.71–0.76), and social (OR: 0.55, 95% CI: 0.53–0.56) living conditions exhibited inverse associations with any alcohol consumption. Similar inverse associations of binge drinking were noted with the economic (OR: 0.84, 95% CI: 0.76–0.92), demographic (OR: 0.91, 95% CI: 0.85–0.98), and social (OR: 0.78, 95% CI: 0.73–0.82) indices.

**Conclusion:**

In general, our findings revealed the associations of alcohol consumption and binge drinking with the regional characteristics of living conditions.

## Background

 Per capita alcohol consumption globally increased from 5.9 L to 6.5 L during 1990–2017 and is projected to reach 7.6 L by 2030^1^. Apparently, the issue is not only in the success/failure of the implementation of legislative regulation of alcohol consumption and the definite existing conflict of interest in alcohol producers.^2,3^ The fact is that alcohol consumption is deeply rooted in the culture and traditions of mankind.^4,5^ As a relatively important component of human life, alcohol consumption depends on various aspects of life rather than legislative norms alone. Numerous studies demonstrated the dependence of alcohol consumption on the social and economic characteristics of residence (1), economic, social, and ethnic inequality (2), the characteristics and infrastructure of residential development (3), and the social norms of the community (4).^6,7^ Understanding the entire environmental context of alcohol consumption can expand the traditional framework of anti-alcohol policy and improve preventive interventions at different territorial and environmental levels.

 For Russia, a territorially large country, the issues of alcohol consumption and alcoholism are quite relevant, despite the fact that over the past two decades, the level of alcohol consumption has significantly declined as a result of the implementation of consistent anti-alcohol policy measures and the general improvement of the social and economic standards of living.^8-10^ Noteworthy regional differences in alcohol consumption are observed in Russia.^11^ Considering that the impact of the environment may be one of the contributing factors to such regional differences, we previously analyzed the dependence of individual alcohol consumption on the characteristics of the regions of residence.^12^ This analysis was performed using the materials of Stage 1 of the nationwide study, Epidemiology of Cardiovascular Diseases and Their Risk Factors in the Regions of the Russian Federation (ESSE-RF), conducted in 2012-2014 in 13 regions of Russia. Later, the study was continued with the participation of 4 and 15 administrative regions of Russia during 2017-2018 (Stage 2) and 2020-2022 (Stage 3), respectively. The use of all three stages of the ESSE-RF study facilitated a significant increase in the analytical sample size and yielded more meaningful results accordingly. It is worth noting that at Stage 1 of the ESSE-RF study, the mean values of specific territorial characteristics were employed for the analysis of the 2012-2014 ESSE-RF data.^12^ In contrast to these short-term territorial characteristics, the recently developed regional indices^13^ empowered us to evaluate the economic, demographic, social, and industrial environmental conditions in Russian regions annually for the period 2005-2022 using a unified methodological approach. All the above-mentioned actions helped in conducting an analytical nationwide study based on 2012-2022 data, aiming to examine the impact of regional living conditions on individual alcohol consumption in Russia.

## Methods

###  Study sample

 The analysis was performed using data from three stages of the Russian nationwide epidemiological study (ESSE-RF):

- ESSE-RF1 was conducted in 13 regions in 2012-2014 and included 21,923 men and women aged 25–64 years.
- ESSE-RF2 was performed in 4 regions during 2017-2018 and encompassed 6,732 men and women aged 25–64 years.
- ESSE-RF3 was implemented in 15 regions in 2020-2022 and consisted of 28,731 men and women aged 35–74 years.

All three stages were conducted using a single methodology for forming a random sample and collecting individual data that were subsequently used in this study. The response rate in all participating regions was at least 70%. The ESSE-RF1^14^ and ESSE-RF3^15^ protocols have already been published. During all three stages, according to healthcare facilities, the Kish method with systematic multistage random sampling based on the territorial principle was employed to form the sample. The study was performed in accordance with the standards of good clinical practice and the principles of the Declaration of Helsinki. In addition, the study protocols were approved by the Ethics Committee of the National Medical Research Center for Preventive Medicine (Moscow). Further, written informed consent was obtained from all participants prior to their inclusion in the study.

 Of the initial 57,386 study participants at all three ESSE-RF stages, 3,484 (6.1% of the sample) had missing data regarding alcohol consumption. The analysis of missing data indicated a significant dependence on the survey wave. In ESSE-RF1, the proportion of missing values was 13.4%, while in ESSE-RF2 and ESSE-RF3, it was 6.0% and only 0.5%, respectively (Supplementary file 1, Table S1). The proportion of missing data was higher among respondents who had low incomes, were single, had no higher education, and were younger and male (*P* = 0.090).

 After excluding these cases, the final analytical sample comprised 53,902 individuals, with 18,992, 6,328, and 28,582 individuals involved in ESSE-RF1 (2012-2014), ESSE-RF2 (2017-2018), and ESSE-RF3 (2020-2022), respectively. Data on the years of data collection, sample sizes, and locations of the regions participating in the study are presented in Supplementary file 1, Table S2.

###  Regional living conditions

 Four regional indices characterizing economic, demographic, social, and industrial ecological aspects, respectively, were employed to define the living conditions of the population in the regions.^13^ Moreover, the index-based assessment was purposefully used because, based on prior experience, it was practically impossible to identify individual regional characteristics as independent predictors due to their complex and sometimes high correlation with each other. For example, if industrial production volumes and environmental pollution are strongly correlated not only with the outcome but also with each other, it is almost impossible to determine which specific indicator is the true predictor. Most likely, both metrics represent the characteristics of some industrial and environmental phenomena that affect the outcomes.

 The values of the regional indices are dimensionless quantities with a normal distribution, a mean of 0, and a standard deviation of 1, calculated for each year using the principal component analysis.

 For the economic index, an increase in the index value indicates growth of the economy, income, household expenditures, and inequality in income distribution. Regarding the demographic index, this increase represents a shift in the age and gender composition toward younger ages and the male population, as well as an increase in the birth rate and population growth. Concerning the social index, it denotes an improvement in the social characteristics of the regions expressed via reductions in the rates of abortions, crime, suicides, and road accidents, as well as a decrease in alcohol sales. For the industrial index, such an increase demonstrates the growth of industrial production volume with a simultaneous deterioration of the environment.

 The annual values of the regional indices were combined with the individual data of the ESSE-RF participants based on the date of the survey and interview. The values of regional indices in the regional samples are provided in Supplementary file 1, Figures S1-S4).

 Studies from countries with regionally differentiated legislation indicated that alcohol policy is one of the most essential predictors of individual alcohol consumption.^6^ Alcohol policy in Russia is mainly formed at the national level and includes both pricing regulations and restrictions on alcohol availability.^10^ Minor regional differences in alcohol policy in this country (mainly regarding the prohibition of sales at certain times) correlate with regional alcohol sales volumes, the indicators that are already incorporated into the social index used in our study. In this regard, alcohol policy indicators were not included in the research analysis.

###  Methods for assessing alcohol consumption and binge drinking

 Data on alcohol consumption were collected from a survey of study participants. The same form of the alcohol consumption questionnaire was utilized at all three stages of the ESSE-RF study. Methodologically, the instrument was the Beverage-Specific Quantity-Frequency (BSQF) questionnaire, which is widely used in population-based studies, particularly across European countries.^16^ The respondents were asked whether they had been consuming alcohol over the last 12 months. If the answer was yes, the respondent was asked to assess the frequency and consumption volume of specific alcoholic beverages. The mean volume of individual consumption was calculated based on the frequency, volume, and type of consumed alcoholic beverages.^12^

 When categorizing the consumption volumes, we were guided by the structure of alcohol consumption obtained in our study and the criteria of moderate drinking by the American National Institute on Alcohol Abuse and Alcoholism (15 and 8 standard drinks for men and women per week, respectively).^17^ Accordingly, two study outcomes were specified for the analysis:

- Alcohol consumption (any alcohol consumption in the past 12 months)
- Binge drinking (alcohol consumption in amounts hazardous to health, specifically ≥ 15 standard drinks for men and ≥ 8 standard drinks for women per week over the past 12 months)

Supplementary file 1, Figure S5 displays the frequencies of any alcohol consumption and binge drinking in the regional samples.

###  Other individual characteristics

 Of the individual variables, socioeconomic and demographic characteristics with the highest level of evidence in terms of their influence on alcohol consumption were assessed, including gender, age, place of residence (urban vs. rural areas), level of education (presence/absence of higher education), marital status (single/married), and income level. Income level was indirectly evaluated based on three questions that characterized the share of income spent on food and a respondent’s opinion regarding the financial well-being of the family and the family wealth compared to other families. An income score ranging from 3 (the poorest) to 15 (the richest) points was used in the statistical analysis.

###  Statistical data analysis

 Differences in categorical variables were analyzed using contingency tables and Pearson’s chi-square test. In addition, logistic regression models were employed to assess the associations. The analyzed data represented a complex two-level sample (individuals were nested in regions). Therefore, generalized estimating equations with robust standard errors were used to evaluate the associations. The associations in our study are expressed as odds ratios (ORs) and 95% confidence intervals (CIs). Initially, a type 1 model was constructed that included only regional indices. Then, individual covariates were introduced into a type 2 model. Given that the level of alcohol consumption by study participants was changing over the duration of the ESSE-RF study,^9^ dummy variables (ESSE-RF1 and ESSE-RF3) were also introduced into a type 2 model. The Akaike information and Bayesian information criteria were utilized to assess the quality of the models. Stratified analyses by all individual variables (gender, age, place of residence, level of education, marital status, and income level), as well as the analysis of the interaction between regional indices, were performed as well. Moreover, effect modification was tested by the likelihood ratio test comparing models with and without a multiplicative interaction term for subsample categories.

 To evaluate the robustness of the obtained results, in addition to the total sample, a separate analysis was conducted only on the ESSE-RF1 and ESSE-RF3 samples.

 Alcohol consumption is traditionally low in a number of Russian regions (the North Caucasian republics of Dagestan, Kabardino-Balkaria, and North Ossetia–Alania).^9^ To assess the possible bias of this pattern on the overall obtained results, an additional analysis was performed, excluding data from these three regions.

 An analysis of the missing data probability from individual and regional characteristics was conducted since there were extensive, non-random missing data (6.1%). Respondents who refuse to answer surveys or have great difficulty agreeing to the survey are usually characterized by a higher frequency and intensity of bad habits, including alcohol consumption.^18,19^ Therefore, an additional analysis of alcohol consumption associations in the full sample (57,386 study participants) with imputed outcomes was performed to evaluate the sustainability of the main results. The missing data for alcohol consumption were reconstructed using the k-nearest neighbor algorithm. Data imputation was conducted based on input parameters (i.e., gender, age, place of residence, level of education, marital status, and income level).

 The Wald chi-squared test was employed to estimate the conditional contribution of individual characteristics and regional indices to alcohol consumption. The critical level of statistical significance was assumed at 0.05. All statistical procedures were performed in SPSS, version 22 (IBM Corporation, USA).

## Results

 The main characteristics of the analytical sample are presented in Table 1. The samples were dominated by urban residents, who were predominantly women, with an average level of income. The majority of them were married and had no higher education. In addition, any alcohol consumption and binge drinking patterns were observed in 68.7% and 4.1% of samples, respectively.

**Table 1:** Main Characteristics of the Analytical Sample

**Characteristics**	**Number (Percent)**
Place of residence	
Urban	42,539 (78.9)
Rural	11,363 (21.1)
Gender	
Men	23,412 (43.4)
Women	30,490 (56.6)
Income	
Low (3-8 points)	15,732 (29.2)
Medium (9-10 points)	28,479 (52.8)
High (11-15 points)	9691 (18.0)
Marital status	
Single	17,617 (32.7)
Married	36,285 (67.3)
Higher education	
No	29,203 (54.2)
Yes	24,699 (45.8)
Age (year)	
25-34	5583 (10.4)
35-44	12,469 (23.1)
45-54	14,107 (26.2)
55-64	15,003 (27.8)
65-75	6740 (12.5)
Any alcohol consumption	
No	16,859 (31.3)
Yes	37,043 (68.7)
Binge drinking	
No	51,690 (95.9)
Yes	2212 (4.1)

###  The main effects of regional indices on alcohol consumption

 The associations of any alcohol consumption and binge drinking with regional living conditions are summarized in Table 2. In the type 1 model, any alcohol consumption was directly associated with the industrial index and inversely associated with the economic, demographic, and social indices. The introduction of individual covariates and the ESSE-RF1 and ESSE-RF3 dummy variables into the type 2 model did not change these associations. An increase in the industrial index by 1 unit yielded an increase in the individual probability of any alcohol consumption by 66% (OR = 1.66, 95% CI: 1.59–1.72).

**Table 2:** Associations of any alcohol consumption and binge drinking with regional living conditions

**Predictors**	**Any alcohol consumption**				**Binge drinking**			
	**Model 1**		**Model 2**		**Model 1**		**Model 2**	
	**OR (95% CI)**	***P *** **value**	**OR (95% CI)**	***Р*** **value**	**OR (95% CI)**	***Р*** **-value**	**OR (95% CI)**	***Р*** **value**
Individual characteristics								
Men (Ref. women)			1.43 (1.37, 1.49)	0.001			3.95 (3.57, 4.37)	0.001
Age (years)			0.98 (0.97, 0.99)	0.001			0.97 (0.96, 0.98)	0.001
Rural (Ref. urban)			0.95 (0.91, 0.99)	0.047			0.88 (0.79, 0.98)	0.005
Marital status (Ref. single)			1.17 (1.12, 1.23)	0.001			0.95 (0.86, 1.05)	0.320
Higher education (Ref. no)			1.25 (1.20, 1.31)	0.001			0.83 (0.75, 0.90)	0.001
Income, points			1.06 (1.05, 1.08)	0.001			1.03 (1.01, 1.06)	0.044
Regional characteristics (indices)								
Economic	0.87 (0.83, 0.90)	0.001	0.75 (0.72, 0.78)	0.001	0.87 (0.80, 0.95)	0.002	0.84 (0.76, 0.92)	0.001
Demographic	0.65 (0.63, 0.67)	0.001	0.74 (0.71, 0.76)	0.001	0.86 (0.80, 0.92)	0.001	0.91 (0.85, 0.98)	0.016
Industrial	1.66 (1.60, 1.72)	0.001	1.66 (1.59, 1.72)	0.001	1.31 (1.22, 1.40)	0.001	1.32 (1.22, 1.42)	0.001
Social	0.58 (0.57, 0.60)	0.001	0.55 (0.53, 0.56)	0.001	0.77 (0.73, 0.81)	0.001	0.78 (0.73, 0.82)	0.001
AIC value	50634.0		47680.5		16562.5		15444.2	
BIC value	49255.4		47467.6		15431.2		14378.8	

*Note*. OR: Odds ratio; CI: Confidence interval; AIC: Akaike information criterion; BIC: Bayesian information criterion. Type 2 models are adjusted for the ESSE-RF1 (2012–2014 survey wave) and ESSE-RF3 (2020–2022 survey wave) dummy variables. ESSE-RF: Epidemiology of Cardiovascular Diseases and Their Risk Factors in the Regions of the Russian Federation.

 Inverse associations were found between any alcohol consumption and the economic (OR = 0.75, 95% CI: 0.72–0.78), demographic (OR = 0.73, 95% CI: 0.71–0.76), and social (OR = 0.55, 95% CI: 0.53–0.56) indices.

 Similarly, binge drinking in the type 1 model was directly related to the industrial index and inversely related to the demographic, economic, and social indices. Based on the results, the introduction of individual covariates and dummy variables (ESSE-RF1 and ESSE-RF3) into the type 2 model failed to alter these associations. An increase in the industrial index by 1 unit could increase the individual probability of binge drinking by 31% (OR = 1.31, 95% CI: 1.22–1.40). Inverse associations were observed between any alcohol consumption and the economic (OR = 0.84, 95% CI: 0.76–0.92), demographic (OR = 0.91, 95% CI: 0.85, 0.98), and social (OR = 0.78, 95% CI: 0.73–0.82) indices.

**Figure 1:**
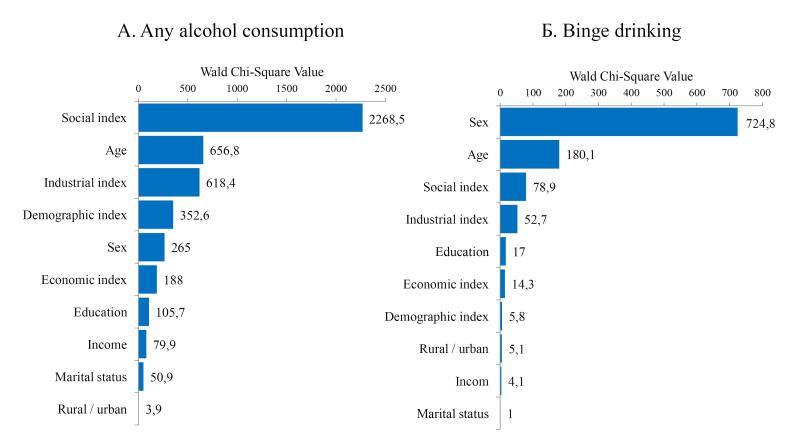
Contribution of individual and regional characteristics to the probability of any alcohol consumption and binge drinking. *Note*. Wald chi-square values represent the relative contribution of each factor to alcohol consumption. In addition, the economic index includes regional characteristics of the economy, household income and expenditure, and inequality in income distribution. Further, the demographic index contains regional characteristics of the age and gender structure, birth rate, and population growth. Moreover, the social index encompasses the regional levels of abortion, crime, suicide, road accidents, and alcohol sales. Finally, the industrial index includes the regional characteristics of industrial production volume and environmental conditions

 Although conventional individual sociodemographic characteristics have been proven to influence alcohol consumption, social living conditions (the social index) make the highest contribution to the probability of any alcohol consumption (Figure 1A). In addition, fairly large contributions are noted for the industrial, demographic, and economic indices (in descending order).

 The contribution of regional living conditions to the probability of binge drinking was significantly lower than that of gender and age (Figure 1B). Nevertheless, the contributions of the social and industrial indices were fairly high, while those of the economic and demographic indices were somewhat lower.

###  The interaction of predictors with regional indices in relation to alcohol consumption

 The analysis of interactions between individual and regional characteristics revealed stronger alcohol consumption associations in specific subgroups. Thus, the stronger associations of any alcohol consumption were typical for women in all regional indices: for urban residents in the industrial index, for single people in the industrial index, and for people with higher education in the industrial and social indices (Table 3). Participants with middle incomes were characterized by the strongest associations in economic and industrial indices, while people with high incomes were characterized by the strongest associations in demographic indices. Among the age-related features, there was a lack of associations according to the economic index in the youngest age group (25–34 years old), as well as a decrease in the level of associations of any alcohol consumption with the demographic index with age.

**Table 3:** Subsample analysis of the association of any alcohol consumption with regional living conditions: type 2 models

**Predictors and subsamples**	**Predictors/Regional indices**							
	**Economic**		**Demographic**		**Industrial**		**Social**	
	**OR (95% CI)**	***Р*** **for interaction**	**OR (95% CI)**	***Р*** **for interaction**	**OR (95% CI)**	***Р*** **for interaction**	**OR (95% CI)**	***Р*** ** for interaction**
Place of residence (rural vs. Ref. urban)		0.410		0.450		0.028		0.190
Rural subsample	0.74 (0.68, 0.82)		0.76 (0.71, 0.82)		1.42 (1.23, 1.64)		0.54 (0.51, 0.57)	
Urban subsample	0.75 (0.71, 0.78)		0.72 (0.70, 0.75)		1.69 (1.62, 1.76)		0.54 (0.53, 0.56)	
Gender (men vs. Ref. women)		0.022		0.001		0.0001		0.0001
Men subsample	0.80 (0.75, 0.85)		0.84 (0.80, 0.88)		1.49 (1.40, 1.59)		0.58 (0.56, 0.60)	
Women subsample	0.70 (0.66, 0.74)		0.64 (0.62, 0.67)		1.81 (1.72, 1.91)		0.51 (0.49, 0.52)	
Income (points)		0.001		0.033		0.012		0.940
3-8 subsample	0.77 (0.71, 0.83)		0.76 (0.72, 0.81)		1.59 (1.48, 1.70)		0.55 (0.53, 0.57)	
9-10 subsample	0.71 (0.67, 0.76)		0.74 (0.71, 0.77)		1.77 (1.67, 1.88)		0.54 (0.52, 0.56)	
11-15 subsample	0.82 (0.73, 0.91)		0.66 (0.60, 0.71)		1.52 (1.38, 1.66)		0.56 (0.53, 0.60)	
Marital status (married vs. Ref. single)		0.390		0.480		0.028		0.110
Single subsample	0.73 (0.69, 0.79)		0.70 (0.66, 0.74)		1.63 (1.53, 1.74)		0.56 (0.54, 0.58)	
Married subsample	0.75 (0.71, 0.79)		0.75 (0.72, 0.78)		1.68 (1.60, 1.77)		0.54 (0.52, 0.55)	
Higher education (yes vs. Ref. no)		0.230		0.064		0.009		0.0001
No subsample	0.78 (0.73, 0.82)		0.73 (0.70, 0.76)		1.57 (1.49, 1.66)		0.57 (0.55, 0.58)	
Yes subsample	0.72 (0.67, 0.76)		0.74 (0.70, 0.77)		1.77 (1.67, 1.88)		0.52 (0.50, 0.54)	
Age (year)		0.001		0.001		0.250		0.130
25-34 subsample	0.98 (0.83, 1.16)		0.59 (0.50, 0.69)		1.31 (1.15, 1.50)		0.64 (0.59, 0.70)	
35-44 subsample	0.71 (0.64, 0.78)		0.73 (0.68, 0.79)		1.75 (1.60, 1.93)		0.51 (0.48, 0.53)	
45-54 subsample	0.74 (0.68, 0.80)		0.69 (0.65, 0.74)		1.71 (1.58, 1.86)		0.55 (0.52, 0.57)	
55-64 subsample	0.75 (0.70, 0.81)		0.75 (0.70, 0.79)		1.68 (1.57, 1.79)		0.56 (0.54, 0.58)	
65-74 subsample	0.74 (0.67, 0.82)		0.84 (0.78, 0.91)		1.27 (1.08, 1.49)		0.49 (0.45, 0.53)	
Economic (interaction)	–	–	0.69 (0.55, 0.87)	0.002	0.83 (0.58, 1.20)	0.330	0.89 (0.77, 1.04)	0.140
Demographic (interaction)	–	–	–	–	1.98 (1.04, 3.79)	0.038	1.00 (0.89, 1.13)	0.970
Industrial (interaction)	–	–	–	–	–	–	1.42 (1.05, 1.92)	0.023

*Note*. OR: Odds ratio; CI: Confidence interval; ESSE-RF: Epidemiology of Cardiovascular Diseases and Their Risk Factors in the Regions of the Russian Federation. Models are adjusted for the ESSE-RF1 (2012–2014 survey wave) and ESSE-RF3 (2020–2022 survey wave) dummy variables.

 The analysis demonstrated only weak interaction effects between individual characteristics and regional factors in relation to binge drinking (Table 4). Rural residents showed stronger associations between binge drinking and economic/industrial indices. Among men, binge drinking was linked to the economic index (while this association was statistically non-significant in women). In individuals with middle and high incomes and those aged 35–44, binge drinking was associated with the social and economic indices, respectively.

**Table 4:** Subsample Analysis of the Association Binge Drinking With Regional Living Conditions: Type 2 Models

**Predictors and subsamples**	**Predictors/Regional indices**							
	**Economic**		**Demographic**		**Industrial**		**Social**	
	**OR (95% CI)**	***Р*** ** for interaction**	**OR (95% CI)**	***Р*** ** for interaction**	**OR (95% CI)**	***Р*** ** for interaction**	**OR (95% CI)**	***Р*** ** for interaction**
Place of residence (rural vs. Ref. urban)		0.002		0.097		0.002		0.730
Rural subsample	0.59 (0.47, 0.73)		1.09 (0.91, 1.32)		2.12 (1.56, 2.87)		0.73 (0.63, 0.84)	
Urban subsample	0.90 (0.82, 1.00)		0.89 (0.82, 0.96)		1.24 (1.14, 1.34)		0.79 (0.75, 0.85)	
Gender (men vs. Ref. women)		0.014		0.0590		0.650		0.063
Men subsample	0.79 (0.71, 0.88)		0.93 (0.85, 1.01)		1.30 (1.19, 1.42)		0.80 (0.75, 0.85)	
Women subsample	0.99 (0.84, 1.20)		0.85 (0.73, 0.99)		1.32 (1.15, 1.51)		0.73 (0.65, 0.82)	
Income (points)		0.088		0.350		0.300		0.001
3-8 subsample	0.81 (0.67, 0.98)		0.91 (0.79, 1.05)		1.24 (1.06, 1.45)		0.88 (0.79, 0.97)	
9-10 subsample	0.81 (0.71, 0.92)		0.94 (0.84, 1.04)		1.41 (1.27, 1.57)		0.73 (0.67, 0.79)	
11-15 subsample	0.97 (0.81, 1.17)		0.79 (0.68, 0.93)		1.23 (1.07, 1.42)		0.75 (0.66, 0.84)	
Marital status (married vs. Ref. single)		0.980		0.100		0.450		0.690
Single subsample	0.86 (0.73, 1.02)		0.98 (0.85, 1.12)		1.23 (1.07, 1.41)		0.80 (0.72, 0.88)	
Married subsample	0.83 (0.75, 0.93)		0.89 (0.82, 0.97)		1.35 (1.24, 1.48)		0.77 (0.72, 0.82)	
Higher education (yes vs. Ref. no)		0.720		0.330		0.510		0.790
No subsample	0.83 (0.73, 0.94)		0.95 (0.87, 1.05)		1.27 (1.14, 1.41)		0.79 (0.73, 0.85)	
Yes subsample	0.85 (0.75, 0.98)		0.87 (0.77, 0.97)		1.35 (1.22, 1.50)		0.76 (0.69, 0.82)	
Age (year)		0.010		0.590		0.750		0.082
25-34 subsample	0.86 (0.65, 1.13)		0.81 (0.62, 1.05)		1.10 (0.91, 1.33)		0.93 (0.80, 1.10)	
35-44 subsample	0.72 (0.61, 0.84)		1.02 (0.93, 1.16)		1.53 (1.34, 1.74)		0.72 (0.65, 0.80)	
45-54 subsample	0.89 (0.75, 1.04)		0.85 (0.75, 0.97)		1.21 (1.05, 1.39)		0.79 (0.71, 0.87)	
55-64 subsample	0.93 (0.75, 1.15)		1.00 (0.84, 1.18)		1.37 (1.16, 1.63)		0.75 (0.66, 0.86)	
65-74 subsample	0.94 (0.64, 1.37)		0.86 (0.64, 1.15)		1.05 (0.59, 1.88)		0.67 (0.48, 0.92)	
Economic (interaction)	–	–	0.85 (0.53, 1.37)	0.510	0.85 (0.65, 1.11)	0.240	0.83 (0.66, 1.04)	0.110
Demographic (interaction)	–	–	–	–	2.66 (1.24, 5.70)	0.012	1.13 (0.75, 1.70)	0.570
Industrial (interaction)	–	–	–	–	–	–	1.85 (1.37, 2.50)	0.001

*Note*. OR: Odds ratio; CI: Confidence interval; ESSE-RF: Epidemiology of Cardiovascular Diseases and Their Risk Factors in the Regions of the Russian Federation. Models are adjusted for the ESSE-RF1 (2012–2014 survey wave) and ESSE-RF3 (2020–2022 survey wave) dummy variables.

 An interaction between the regional indices was found in addition to individual predictors (Supplementary file 1, Tables 3 and 4). For any alcohol consumption and binge drinking, a significant interaction was detected between the demographic and industrial indices (OR = 1.98: 1.04–3.79 and OR = 2.66: 1.24–5.70, respectively). More precisely, an increase in one index reduced (or even reversed) the strength of the association of the other index with alcohol consumption. Thus, when the demographic index values were below the median, the association of the industrial index corresponded to the main effect for both any alcohol consumption (OR = 1.59, 95% CI: 1.38–1.83) and binge drinking (OR = 1.63, 95% CI: 1.35–2.00). However, at demographic index values above the median, the association of the industrial index with any alcohol consumption became non-significant (OR = 1.06, 95% CI: 0.74–1.50). At the same time, its association with binge drinking changed to an association opposite to the main effect (OR = 0.60, 95% CI: 0.36–0.98).

 A similar direct interaction, observed for both any alcohol consumption and binge drinking, was found between industrial and social indices (OR = 1.42, 95% CI: 1.05–1.92 and OR = 1.85, 95% CI: 1.37–2.50, respectively). Specifically, this interaction was manifested by stronger associations of the social index with both outcomes at lower values of the industrial index.

 There was an inverse interaction between the economic and demographic indices regarding any alcohol consumption (OR = 0.69, 95% CI: 0.55–0.87), indicating that the association of one index strengthened with increasing values of the other index. Hence, the association of the demographic index with any alcohol consumption was statistically non-significant when economic index values were less than the median (OR = 1.00, 95% CI: 0.79–1.27). In addition, for economic index values above the median, the association corresponded to the main effect (OR = 0.67, 95% CI: 0.56–0.80).

###  Analysis of association stability 

 A separate analysis of the ESSE-RF1 (2012-2014) and ESSE-RF3 (2020-2022) samples yielded results similar to those for the total sample (Supplementary file 1, Table S3).

 Excluding three regions with traditionally (culturally) low levels of alcohol consumption from the analysis did not change the direction and level of statistical significance of the associations with any alcohol consumption and binge drinking (Supplementary file 1, Table S4).

 The probability of missing data on alcohol consumption in the multivariate analysis was dependent on a number of individual and regional characteristics (Supplementary file 1, Table S5). In particular, the probability of missing data decreased with higher economic index values but increased with higher demographic and social index values. However, the analysis of the full sample, with imputed data on alcohol consumption, did not alter the direction or statistical significance of the associations with any alcohol use and binge drinking (Supplementary file 1, Table S6).

## Discussion

 The deterioration of economic and social characteristics in the studied regions, the demographic hole in the regions, and the growth of industrial development increased the likelihood of individual alcohol consumption. These results for all three stages of the ESSE-RF study are in line with the data obtained in a previously conducted analysis solely for ESSE-RF1.^12^ In the current study, more pronounced and steady associations, as well as the detection of associations of alcohol consumption with economic characteristics in the regions, are explained using more valid annual regional indices and an extremely larger sample size.

 The most pronounced associations of individual alcohol consumption were found with the social characteristics of the regions. The social index included the sales of beer and strong spirits (the most popular types of alcoholic beverages in Russia), which largely explains the identified associations.

 Eventually, if the entire population of a region actively purchases alcoholic beverages, then it is obvious that the probability of alcohol consumption is higher in any specific individual from this population compared to regions with low sales. On the other hand, the more alcohol is consumed by others, the more acceptable alcohol consumption becomes for everyone. It is noteworthy that the acquired habits of the majority of the population are, in a sense, projected onto each individual in the entire population of this region. From the standpoint of behavioral psychology, this issue is consistent with Isaac Ajzen’s theory of planned behavior. According to this theory, an individual’s intentions and behavior are largely the result of a combination of attitudes toward behavior, subjective norms, and perceived behavioral control.^20^ With regard to alcohol consumption volume, the determinacy of the behavioral act by subjective and descriptive (including social) norms is quite high^21^. Likewise, other population-based studies confirmed steady associations existing between the population and individual levels of alcohol consumption.^22-24^ The cross-sectional design of this study has limited the interpretation of individual alcohol consumption due to the high prevalence of alcohol use in the region. In our opinion, population prevalence and individual alcohol consumption mutually influence each other. In other words, the probability of individual consumption is higher when people consume alcohol more. Conversely, individual drinking behavior contributes to the overall population prevalence. Undoubtedly, this hypothesis requires further in-depth analysis with an appropriate study design.

 In addition to the levels of alcohol sales, the social index includes other regional characteristics that can determine the individual probability of alcohol consumption. At the level of small territories, the probability of alcohol consumption and binge drinking increases in socially disadvantaged areas and neighborhoods,^25,26^ despite the fact that alcohol consumption at the population level is often not included in the area-based deprivation indices. Consequently, it can be assumed that population alcohol consumption is an important factor (even though it is just one of the many constituent characteristics of the social well-being of the population), determining the individual risks of alcohol consumption.

 The direct dependence of alcohol consumption on the industrial development of regions may be a consequence of the formation of a subculture involving high consumption of alcoholic beverages among the working class in such regions. On the one hand, masculinity is conventionally associated with alcohol consumption.^27^ On the other hand, the proportion of those who consume and abuse alcohol is higher in traditionally male-dominated professions, which include industrial workers.^28,29^ Russian studies have also indicated higher volumes of alcohol consumption by industrial workers. The latter generally included unskilled workers, as well as workers engaged in manual labor or those who used machines and mechanisms.^30,31^ A Finnish study conducted at the regional level demonstrated that a high proportion of manual workers in the professional structure of a region was characterized by an increase in individual mortality associated with alcohol.^32^ Apparently, similar to the social index, a projection of the habits of a significant part of the population onto the entire population in that region is observed in the case of the associations between the industrial index and alcohol consumption. In this regard, the inversion of the binge drinking association found in the interaction between the demographic and industrial indices is interesting; industrial development in a region with demographic depression potentiates binge drinking. However, it conversely reduces the likelihood of binge drinking in a region with demographic progressiveness. It is possible that in this case, the demographic structure of society characterizes the type and sector of regional industries, leading to the formation of specific professional communities with different social and cultural traditions. Certainly, the identified interaction requires further in-depth investigation that takes into account the specific characteristics of industrial production, including the type and sector of industry, manufactured products, corporate health promotion policies in industrial settings, and features of technological regulations, as well as how these industrial parameters combine with the demographic structure of the region.

 The demographic and economic indices demonstrate fewer steady associations and a smaller contribution to individual risks of alcohol consumption. It is suggested that demographic index associations be implemented by various mechanisms, which are quite complex in nature. It is thought that the main mechanism of this kind is interregional migration in search of better prospects for life and financial well-being, leading to the occurrence of the social and/or professional mobility of the active part of the population.^33-35^ Due to the outflow of the socially and professionally active part of the working population, the proportion of elderly people and young people with a low level of social and professional mobility is increasing, resulting in the formation of a demographic hole in the region. The individual probability of alcohol consumption in such demographically depressed regions increases since low social and professional mobility is associated with low commitment to health-oriented behavior.^36-38^

 The direct influence of economic characteristics in the region on individual alcohol consumption is intuitively clear. However, the low contribution of the economic index to individual alcohol consumption, according to our data, is consistent with the findings of another study, demonstrating the uncertainty of the effect of some economic characteristics and income inequality in the population.^6^

 The analysis of the interactions between individual and regional predictors revealed several nuances related to alcohol consumption. This was manifested by different expressions of the influence of regional living conditions for individual socio-demographic categories of the population, despite similar overall trends across strata. The most stable interaction effects were identified regarding gender; stronger associations between economic, demographic, industrial, and social conditions with any alcohol consumption, as well as between demographic and social conditions with binge drinking, were observed among women. It is assumed that this may be due to the fact that traditionally social norms are more significant for women than for men.^39,40^ Women perceive alcohol consumption within the framework of severe social punishment since alcohol consumption and alcoholism are considered to be against the desired feminine traits and traditional woman roles in the family and childrearing. Therefore, regional living conditions, which largely characterize social norms or their markers, are more strongly correlated with the likelihood of alcohol consumption among women. Other individual predictors (i.e., income level, education, age, and urban or rural residence) interact with regional indices less significantly; however, the reasons for these interactions are similar, including the differentiation of the importance of social norms and preferences among socio-demographic strata of society.

 When comparing our results with those of other similar studies, it should be noted that there are few such studies. In a 2023 systematic review^6^ (including 81 studies, predominantly conducted in the United States, Australia, and Western Europe) devoted to the evaluation of the associations between regional characteristics and individual probability of alcohol consumption, most studies focused on normative, legal, and pricing policies related to alcohol as regional factors. Few studies analyzed regional characteristics similar to the Russian regional indices used in this study, including per capita alcohol sales, population-level drinking prevalence, crime rates, and income inequality. In general, the results of these studies are consistent with our data, indicating that our findings may be extrapolated to countries with similar socio-economic and cultural characteristics. Nonetheless, the lack of studies in countries with different cultures regarding alcohol consumption limits the extrapolation of our associations to all countries.

 As for the limitations of our study, it should be noted that the ESSE-RF3 stage coincided with the coronavirus disease 2019 pandemic. Considering that the restrictions and isolation of the population introduced during this period affected alcohol consumption,^41^ there could be a possible bias in the results of that study stage. Although separate analyses in the subsamples of stages 1 and 3 of the ESSE-RF study demonstrated trends similar to the total sample, the associations of alcohol consumption with the demographic index in the ESSE-RF3 subsample became statistically insignificant. Probably, the coronavirus disease 2019 pandemic has become a novel, relatively important factor in alcohol consumption, somewhat weakening the habitual associations of living conditions of the population in the regions with alcohol consumption.

 As a limitation of our study, it is necessary to note its cross-sectional design, which has limited the interpretation of the results in terms of causal dependence.

 Another limitation was the use of a self-assessment method of alcohol consumption in the study. This approach is considered an acceptable screening method.^16^ Typically, the self-reported method leads to the underestimation of alcohol consumption volumes; nonetheless, the BSQF approach, which collects detailed information on types and amounts of alcoholic beverages consumed, partially corrects this underestimation^42^. Despite the many advantages of the applied BSQF method, it is noteworthy that the questionnaire is less reliable when measuring irregular patterns of alcohol consumption. In addition, BSQF does not take into account the simultaneous single consumption of various types of alcoholic beverages since the interviewer asks separate questions about each drink. With regard to the conducted study, it is possible to assume a bias in the results if the regularity and/or combination of alcohol consumption is associated with regional living conditions. It is impossible to verify this assumption based on the available data.

 The present study took into account the most traditionally considered individual predictors of alcohol consumption. However, it is worth mentioning that factors such as mental health, employment type, genetics, family traditions, and immediate social circle were not included in the analysis, which can potentially lead to distorted associations.

**Highlights**

- Regional conditions are associated with individual alcohol consumption in Russia.
- Industry, poor economy, demography, and social conditions increase alcohol consumption.
- The strength of associations varies across certain socio-demographic subgroups.

## Conclusion

 The results of our study demonstrated the associations of alcohol consumption and binge drinking with regional characteristics of living conditions. For any alcohol consumption, multiple interactions were observed between individual characteristics and regional living conditions. More precisely, stronger associations were found among women, participants with middle and high incomes, and those with higher education. In contrast, only a tendency for stronger associations with regional characteristics could be identified among rural residents. The revealed different mechanisms may account for these associations. Our findings for Russia are consistent with those of other studies conducted in different countries of the world. Additionally, our results complement the already known theoretical patterns from the standpoint of an integrated approach to assessing the human environment. The practical significance of understanding the conditions for the formation of alcohol consumption levels depending on environmental factors provides additional tools for healthcare managers in terms of differentiating developed preventive approaches and assessing their effectiveness.

## Acknowledgments

 The authors are sincerely grateful to those who assisted in the study and data collection: S. A. Boytsov, V. A. Kutsenko, S. E. Evstifeyeva, A. V. Kapustina, N. S. Karamnova, G. A. Muromtseva, A. A. Keskinov, A. Yu. Yakovchik, and S. M. Yudin (all from Moscow); E. V. Shlyakhto, A. O. Konradi, and O. P. Rotar (all from St. Petersburg); N. V. Kulakova, V. A. Nevzorova, N. V. Shestakova, M. V. Mokshina, and L. V. Rodionova (all from Primorsky Krai); G. V. Tolparov (Republic of North Ossetia–Alania); V. A. Ilyin, A. A. Shabunova, K. N. Kalashnikov, O. N. Kalachikova, and A. V. Popov (all from Vologda Oblast); S. V. Nedogoda, E. V. Chumachek, and A. A. Ledyaeva (all from Volgograd Oblast); G. I. Furmenko, T. M. Chernykh, V. V. Ovsyannikova, and L. V. Bondartsov (all from Voronezh Oblast); O. A. Belova, S. V. Romanchuk, O. A. Nazarova, and O. A. Shutemova (all from Ivanovo Oblast); O. L. Barbarash, G. V. Artamonova, E. V. Indukayeva, and T. A. Mulerova (all from Kemerovo Oblast); Yu. I. Grinshtein, M. M. Petrova, L. K. Danilova, A. A. Evsyukov, V. V. Shabalin, R. R. Ruf, A. A. Kosinova, I. V. Filonenko, and O. A. Baykova (all from Krasnoyarsk Krai); R. A. Libis, E. A. Lopina, I. R. Basyrova, V. N. Nikulin, O. R. Aslyamov, and G. V. Khokhlova (all from Orenburg Oblast); D. V. Duplyakov, S. A. Gudkova, and N. A. Cherepanova (all from Samara Oblast); I. A. Trubacheva, V. S. Kaveshnikov, R. S. Karpov, and V. N. Serebryakova (all from Tomsk Oblast); A. Yu. Efanov, I. V. Medvedeva, M. A. Storozhok, and S. V. Shalayev (all from Tyumen Oblast); I. A. Viktorova, M. A. Livzan, I. A. Grishechkina, and M. Yu. Rozhkova (all from Omsk Oblast); N. N. Prishchepa, N. N. Vezikova, and I. S. Skopets (all from Republic of Karelia); A. N. Redko, S. N. Alekseenko, and S. V. Gubarev (all from Krasnodar Krai); S. S. Yakushin, E. V. Filippov, N. V. Dobrynina, N. N. Nikulina, K. G. Pereverzeva, and K. A. Moseychuk (all from Ryazan Oblast); T. V. Repkina and T. O. Gonoshilova (all from Altai Krai); A. V. Kudryavtsev, N. I. Belova, and L. L. Shagrov (all from Arkhangelsk Oblast); M. A. Samotrueva, A. L. Yasenyavskaya, and E. N. Chernysheva (all from Astrakhan Oblast); S. V. Glukhovskaya, I. A. Levina, and E. A. Shirshova (all from Sverdlovsk Oblast); E. B. Dorzhieva and E. Z. Urbanova (all from Republic of Buryatia); N. Yu. Borovkova, V. K. Kurashin, and A. S. Tokareva (all from Nizhny Novgorod Oblast); Yu. I. Ragino, G. I. Simonova, and A. D. Khudyakova (all from Novosibirsk Oblast); A. V. Solovyova, A. A. Rodionov, and O. V. Kryachkova (all from Tver Oblast); Yu. Yu. Shamurova, I. V. Tantsyreva, and I. N. Baryshnikova (all from Chelyabinsk Oblast); M. G. Ataev, M. O. Radjabov, and M. M. Isakhanova (all from the Republic of Dagestan); M. A. Umetov, L. V. Elgarova, and I. A. Khakuasheva (all from the Republic of Kabardino-Balkaria); E. I. Yamashkina, M. V. Esina, and T. A. Kunyaeva (all from the Republic of Mordovia); A. M. Nikitina, N. V. Savvina, and Yu. E. Spiridonova (all from the Republic of Sakha, Yakutia); E. A. Naumova (the Republic of Chuvashia).

## Competing Interests

 The authors have no conflict of interests associated with the material presented in this manuscript.

## Ethical Approval

 The study protocols were approved by the Ethics Committee of the National Medical Research Center for Preventive Medicine, Moscow, Russia [No. 07-03/12 from 03.07.2012 (ESSE-RF1), No. 03-01/17 from 18.04.2017 (ESSE-RF2), and No. 01-01/20 from 04.02.2020 (ESSE-RF3)].

## Funding

 This study received financial support from the National Medical Research Center for Preventive Medicine, Moscow, Russia.

## Supplementary Files

Supplementary file 1 contains Tables S1-S6 and Figures S1-S5.
