# Supplementary material for: Individual Alcohol Consumption Depending on Regional Living Conditions: Results of a Russian Nationwide Study Based on 2012-2022 Data
Source: J Res Health Sci. 2025 Oct 18;26(1):e00669. doi: 10.34172/jrhs.11352 (PMC12681061; doi:10.34172/jrhs.11352)

JRHS
Journal of Research in Health Sciences
doi:10.34172/jrhs.11352
JRHS 2026; 26(1):e00669-S1

Supplementary file 1

Table S1: Missing data on alcohol consumption by study wave and individual characteristics.

		Alcohol consumption data omission		
**Characteristic**	**Total**	**Number**	**Percent**	**P-value**
Survey wave				0.001
ESSE-RF1 2012–2014	21,923	2931	13.4	
ESSE-RF2 2017–2018	6732	404	6.0	
ESSE-RF3 2020–2022	28,731	149	0.5	
Place of residence				0.770
Rural	12,091	728	6.0	
Urban	45,295	2756	6.1	
Sex				0.090
Men	24,876	1464	5.9	
Women	32,510	2020	6.2	
Income, points				0.001
3-8	16,997	1265	7.4	
9-10	30,190	1711	5.7	
11-15	10,199	508	5.0	
Marital status				0.001
Single	18,889	1272	6.7	
Married	38,497	2212	5.7	
Higher education				0.001
No	31,210	2007	6.4	
Yes	26,176	1477	5.6	
Age (yr)				0.001
25-34	6276	693	11.0	
35-44	13,221	752	5.7	
45-54	15,079	972	6.4	
55-64	16,034	1031	6.4	
65-74	6776	36	0.5	

Table S2: Years of data collection, sample sizes and location of regions participating in the analytical sample of the ESSE-RF study

Region	Federal District	Stage of the ESSE-RF study	Years of data collection	Sample size
Republic of Kabardino-Balkaria	North Caucasian	3	2020-2021	1746
Republic of Buryatia	Far Eastern	3	2020-2021	1791
Republic of Dagestan	North Caucasian	3	2020-2022	2965
Republic of Karelia	Northwestern	2	2017	1490
Republic of Mordovia	Volga	3	2020	1794
Republic of Sakha (Yakutia)	Far Eastern	3	2020-2022	1815
Republic of North Ossetia–Alania	North Caucasian	1	2012-2013	1548
Republic of Chuvashia	Volga	3	2020-2022	1830
Altai Krai	Siberian	3	2021	1900
Krasnodar Krai	Southern	2	2017	1735
Krasnoyarsk Krai	Siberian	1	2014	1461
Primorsky Krai	Far Eastern	1	2013-2014	1707
Arkhangelsk Oblast	Northwestern	3	2021	1808
Astrakhan Oblast	Southern	3	2021	908
Volgograd Oblast	Southern	1	2012-2013	1058
Vologda Oblast	Northwestern	1	2013-2014	1457
Voronezh Oblast	Central	1	2012-2013	1482
Ivanovo Oblast	Central	1	2012-2013	1685
Kemerovo Oblast	Siberian	1	2013	1586
Nizhny Novgorod Oblast	Volga	3	2021	2509
Novosibirsk Oblast	Siberian	3	2021-2022	1199
Omsk Oblast	Siberian	2	2017	1509
Orenburg Oblast (ESSE-RF1)	Volga	1	2012	1352
Orenburg Oblast (ESSE-RF3)	Volga	3	2020-2022	2063
Ryazan Oblast	Central	2	2017-2018	1594
Samara Oblast	Volga	1	2012-2013	1505
Sverdlovsk Oblast	Ural	3	2020-2021	1746
Tver Oblast	Central	3	2020-2021	2011
Tomsk Oblast	Siberian	1	2012	1416
Tyumen Oblast	Ural	1	2012-2013	1497
Chelyabinsk Oblast	Ural	3	2021	2497
City of Saint Petersburg	Northwestern	1	2012-2013	1238

Table S3: Associations of any alcohol consumption and binge drinking with regional living conditions in the subsample excluding the republics of Dagestan, Kabardino-Balkaria, and North Ossetia–Alania: type 2 models.

	**Any alcohol consumption**		**Binge drinking**	
**Predictors**	**OR (95% CI)**	**P-value**	**OR (95% CI)**	**P-value**
Individual characteristics				
Men (ref. Women)	1.29 (1.23, 1.35)	0.001	3.82 (3.45, 4.24)	0.001
Age, years	0.97 (0.96, 0.98)	0.001	0.97 (0.96, 0.98)	0.001
Rural (ref. Urban)	0.94 (0.89, 0.99)	0.013	0.89 (0.79, 0.99)	0.047
Marital status (ref. Single)	1.20 (1.14, 1.25)	0.001	0.96 (0.87, 1.06)	0.410
Higher education (ref. No)	1.25 (1.19, 1.31)	0.001	0.84 (0.77, 0.92)	0.001
Income, points	1.06 (1.04, 1.08)	0.001	1.04 (1.01, 1.07)	0.015
Regional characteristics (indices)				
Economic	0.75 (0.72, 0.78)	0.001	0.86 (0.78, 0.94)	0.001
Demographic	0.76 (0.73, 0.80)	0.001	0.82 (0.75, 0.90)	0.001
Industrial	1.61 (1.54, 1.69)	0.001	1.41 (1.29, 1.53)	0.001
Social	0.58 (0.55, 0.60)	0.001	0.69 (0.63, 0.75)	0.001

Note: Models are adjusted for the ESSE-RF1 (2012–2014 survey wave) and ESSE-RF3 (2020–2022 survey wave) dummy variables

Table S4: Associations between individual and regional predictors and the likelihood of missing alcohol consumption data: type 2 models.

Predictors	Alcohol consumption data omission	
	**OR (95% CI)**	**P-value**
Individual characteristics		
Men (ref. Women)	1.19 (1.09, 1.29)	0.001
Age, years	0.99 (0.98, 0.99)	0.041
Rural (ref. Urban)	1.17 (1.06, 1.29)	0.002
Marital status (ref. Single)	0.91 (0.84, 0.99)	0.028
Higher education (ref. No)	0.93 (0.85, 1.01)	0.080
Income, points	0.97 (0.95, 0.99)	0.012
Regional characteristics (indices)		
Economic	0.76 (0.69, 0.84)	0.001
Demographic	1.35 (1.25, 1.46)	0.001
Industrial	1.02 (0.96, 1.09)	0.480
Social	1.48 (1.42, 1.55)	0.001

Note: Models are adjusted for the ESSE-RF1 (2012–2014 survey wave) and ESSE-RF3 (2020–2022 survey wave) dummy variables

Table S5: Associations of any alcohol consumption and binge drinking with regional living conditions in the full sample: type 2 models.

Predictors	Any alcohol consumption		Binge drinking	
	**OR (95% CI)**	**P-value**	**OR (95% CI)**	**P-value**
Individual characteristics				
Men (ref. Women)	1.39 (1.33, 1.45)	0.001	3.94 (3.57, 4.35)	0.001
Age, years	0.98 (0.97, 0.99)	0.001	0.97 (0.96, 0.98)	0.001
Rural (ref. Urban)	1.00 (0.95, 1.05)	0.990	0.88 (0.78, 0.98)	0.020
Marital status (ref. Single)	1.29 (1.24, 1.34)	0.001	0.95 (0.86, 1.05)	0.340
Higher education (ref. No)	1.28 (1.23, 1.33)	0.001	0.84 (0.77, 0.92)	0.001
Income, points	1.05 (1.03, 1.06)	0.001	1.03 (1.01, 1.06)	0.031
Regional characteristics (indices)				
Economic	0.76 (0.73, 0.79)	0.001	0.84 (0.77, 0.92)	0.001
Demographic	0.74 (0.71, 0.76)	0.001	0.91 (0.84, 0.98)	0.011
Industrial	1.52 (1.46, 1.57)	0.001	1.31 (1.21, 1.41)	0.001
Social	0.57 (0.55, 0.58)	0.001	0.75 (0.71, 0.80)	0.001

Note: Models are adjusted for the ESSE-RF1 (2012–2014 survey wave) and ESSE-RF3 (2020–2022 survey wave) dummy variables

Table S6: Associations of any alcohol consumption and binge drinking with regional living conditions in the ESSE-RF1 subsample (2012-2014) and in the ESSE-RF3 subsample (2020-2024): type 2 models.

	Any alcohol consumption				Binge drinking			
	ESSE-RF1		ESSE-RF3		ESSE-RF1		ESSE-RF3	
**Predictors**	**OR (95% CI)**	**P-value**	**OR (95% CI)**	**P-value**	**OR (95% CI)**	**P-value**	**OR (95% CI)**	**P-value**
Individual characteristics								
Men (ref. Women)	1.21 (1.11, 1.32)	0.001	1.59 (1.50, 1.68)	0.001	3.94 (3.40, 4.56)	0.001	4.59 (3.90, 5.40)	0.001
Age, years	0.98 (0.97, 0.99)	0.001	0.97 (0.96, 0.98)	0.001	0.98 (0.97, 0.99)	0.001	0.96 (0.95, 0.97)	0.001
Rural (ref. Urban)	1.09 (0.99, 1.20)	0.092	0.90 (0.85, 0.96)	0.002	0.87 (0.72, 1.04)	0.130	0.93 (0.79, 1.10)	0.380
Marital status (ref. Single)	1.15 (1.06, 1.25)	0.001	1.18 (1.11, 1.24)	0.001	0.87 (0.75, 1.01)	0.071	0.93 (0.80, 1.09)	0.380
Higher education (ref. No)	1.21 (1.11, 1.31)	0.001	1.30 (1.23, 1.38)	0.001	0.80 (0.70, 0.93)	0.003	0.82 (0.71, 0.94)	0.004
Income, points	1.08 (1.06, 1.11)	0.001	1.06 (1.04, 1.08)	0.001	1.05 (1.01, 1.10)	0.013	1.01 (0.97, 1.06)	0.510
Regional characteristics (indices)								
Economic	0.90 (0.83, 0.98)	0.015	0.69 (0.66, 0.73)	0.001	0.77 (0.67, 0.89)	0.001	0.87 (0.77, 0.99)	0.032
Demographic	0.66 (0.61, 0.70)	0.001	0.81 (0.77, 0.84)	0.001	0.91 (0.78, 1.06)	0.220	0.98 (0.89, 1.08)	0.740
Industrial	1.47 (1.38, 1.55)	0.001	1.46 (1.34, 1.59)	0.001	1.29 (1.17, 1.42)	0.001	1.22 (1.01, 1.47)	0.039
Social	0.63 (0.61, 0.66)	0.001	0.46 (0.44, 0.47)	0.001	0.83 (0.76, 0.91)	0.001	0.67 (0.61, 0.74)	0.001

Note: ESSE-RF1, 2012–2014 survey wave; ESSE-RF3, 2020–2022 survey wave

Figure S1: The value of Economic index in samples of regions participating in the ESSE-RF study.

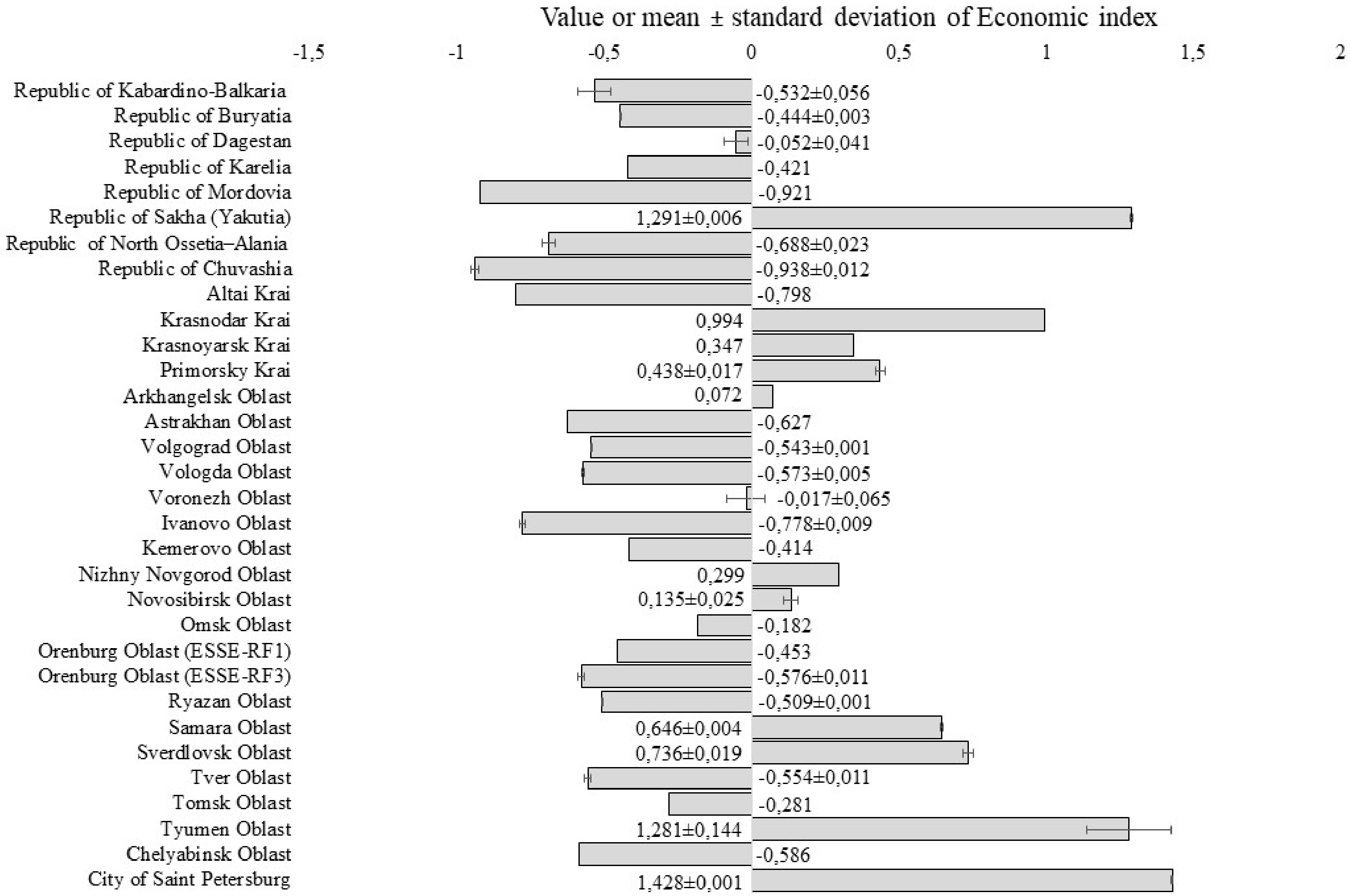

Figure S2: The value of Demographic index in samples of regions participating in the ESSE-RF study.

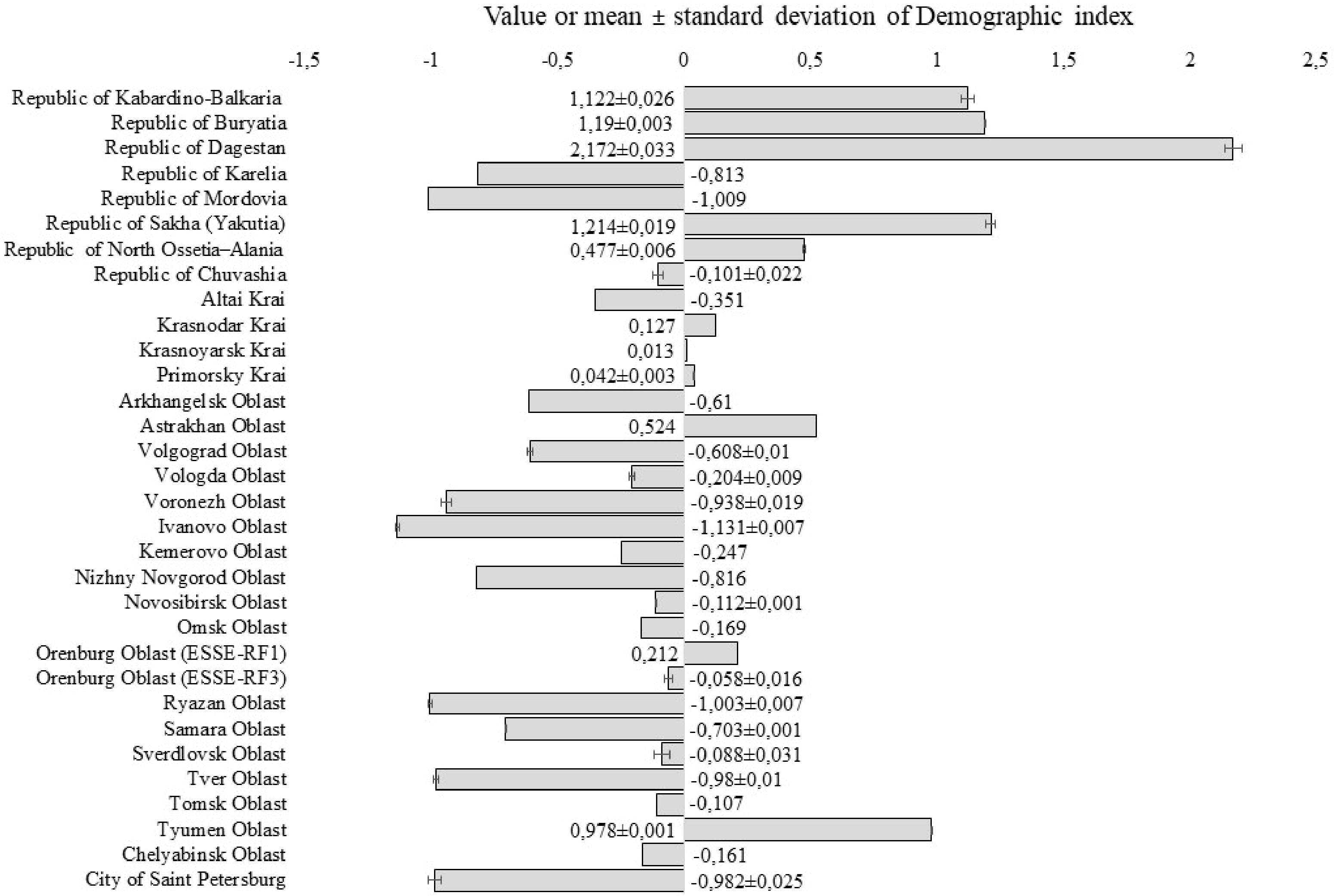

Figure S3: The value of Industrial index in samples of regions participating in the ESSE-RF study.

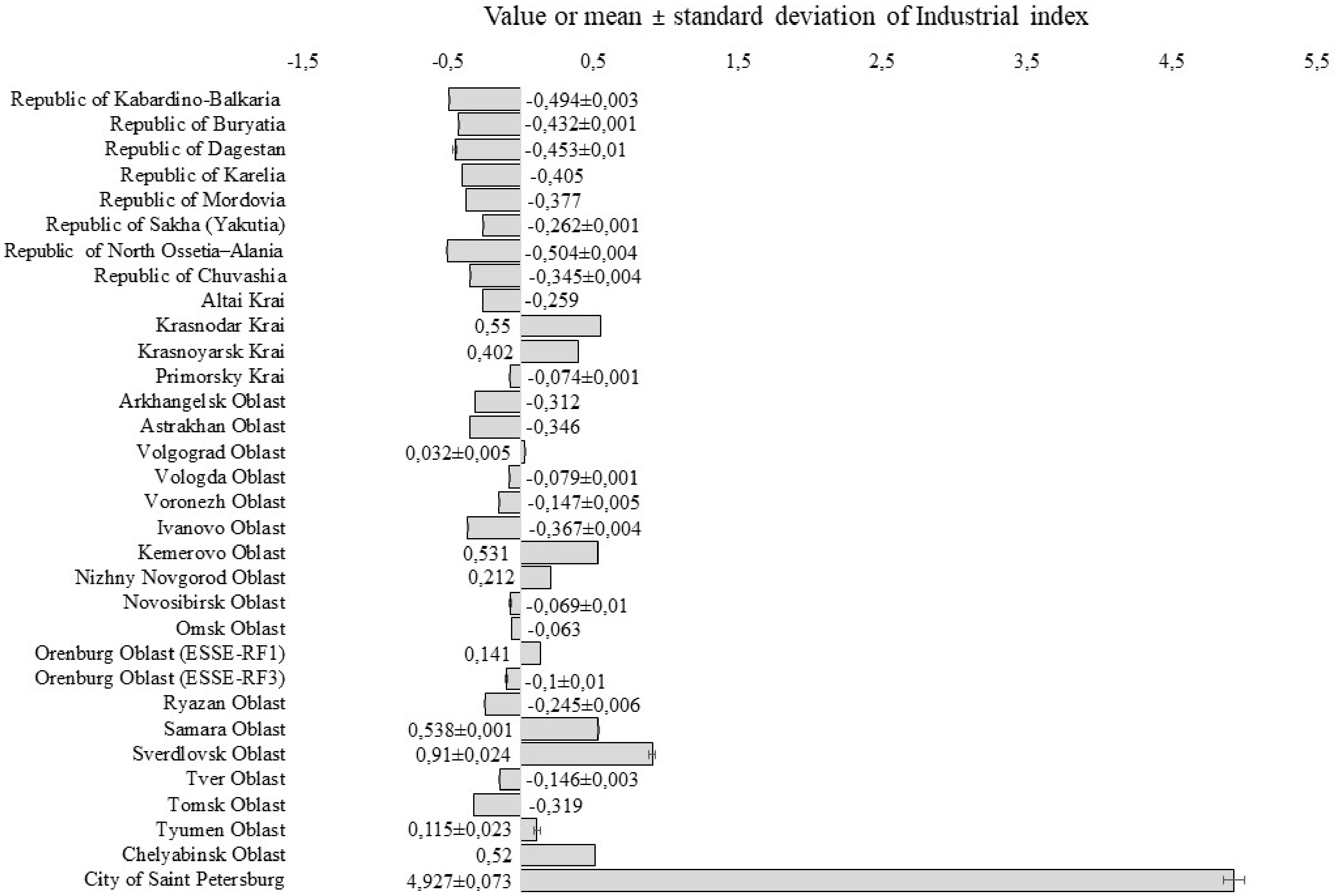

Figure S4: The value of social index in samples of regions participating in the ESSE-RF study.

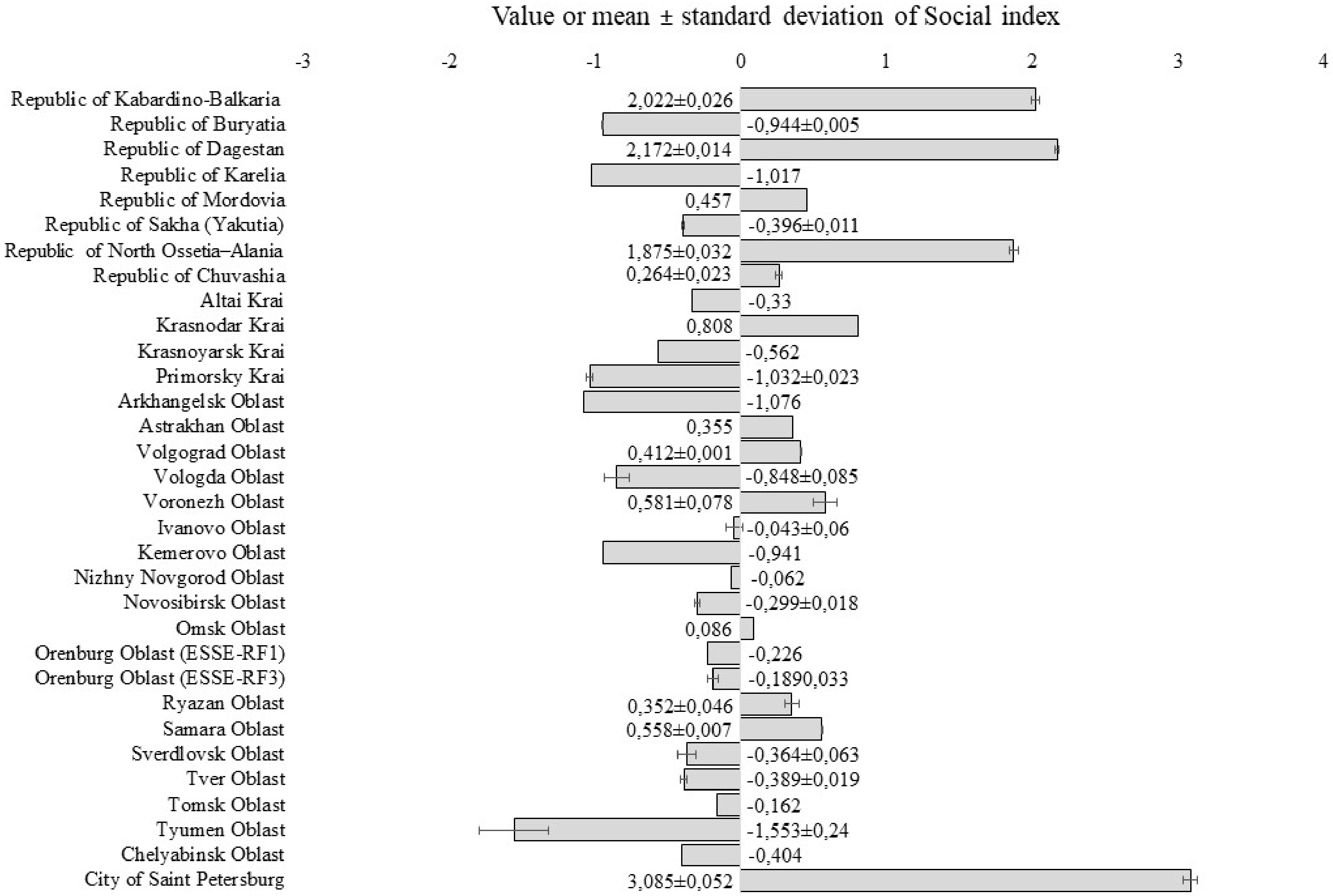

Figure S5: The frequency of alcohol consumption and binge drinking in samples of regions participating in the ESSE-RF study.

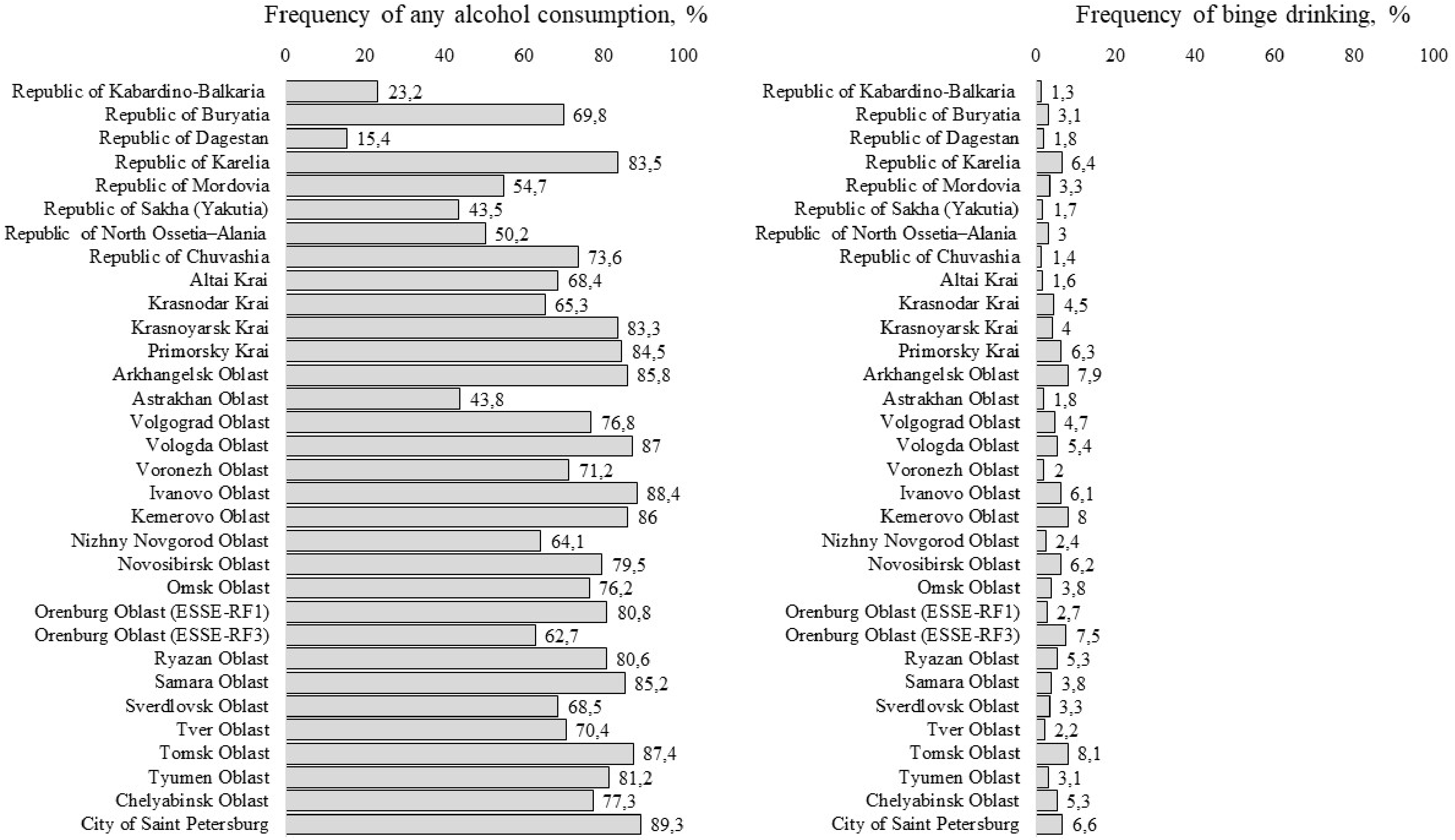

Supplement: Supplementary file 1 — contains Tables S1-S6 and Figures S1-S5. [file jrhs-26-e00669-s001.pdf]
